# SARS-CoV-2 Vaccine Induced Atypical Immune Responses in Antibody Defects: Everybody Does their Best

**DOI:** 10.1007/s10875-021-01133-0

**Published:** 2021-10-20

**Authors:** Ane Fernandez Salinas, Eva Piano Mortari, Sara Terreri, Concetta Quintarelli, Federica Pulvirenti, Stefano Di Cecca, Marika Guercio, Cinzia Milito, Livia Bonanni, Stefania Auria, Laura Romaggioli, Giuseppina Cusano, Christian Albano, Salvatore Zaffina, Carlo Federico Perno, Giuseppe Spadaro, Franco Locatelli, Rita Carsetti, Isabella Quinti

**Affiliations:** 1 Department of Molecular Medicine, Sapienza University of Rome, Viale Dell’Università, 37, Rome, Italy; 2 Diagnostic Immunology Research Unit, Multimodal Medicine Research Area, Bambino Gesù Children’s Hospital, IRCCS, Viale Di San Paolo, 15, Rome, Italy; 3 Department Onco-Haematology, and Cell and Gene Therapy, Bambino Gesù Children Hospital, IRCCS, Rome, Italy; 4 Department of Clinical Medicine and Surgery, University of Naples Federico II, Naples, Italy; 5 Regional Reference Centre for Primary Immune Deficiencies, Azienda Ospedaliera Universitaria Policlinico Umberto I, Rome, Italy; 6 Occupational Medicine/Health Technology Assessment and Safety Research Unit, Clinical-Technological Innovations Research Area, Bambino Gesù Children’s Hospital, IRCCS, Viale di San Paolo, 15, Rome, Italy; 7 Health Directorate, Bambino Gesù Children’s Hospital, IRCCS, Piazza Sant’Onofrio, 4, Rome, Italy; 8 Multimodal Medicine Research Area, Bambino Gesù Children’s Hospital, IRCCS, Piazza Sant’Onofrio, 4, Rome, Italy; 9 Microbiology and Diagnostic Immunology Unit, Bambino Gesù Children’s Hospital, IRCCS, Piazza Sant’Onofrio, 4, Rome, Italy; 10 Department of Translational Medical Sciences, University of Naples Federico II, 80131 Naples, Italy; 11 Dipartimento Materno-Infantile E Scienze Urologiche, Sapienza University of Rome, Rome, Italy; 12 Diagnostic Immunology Clinical Unit, Bambino Gesù Children’s Hospital, IRCCS, Viale Di San Paolo,15, Rome, Italy

**Keywords:** Primary antibody deficiencies, Common variable immune deficiencies, X-linked agammaglobulinemia, COVID-19, SARS-CoV-2, BNT162b2 vaccine, Memory cells, Spike protein, Receptor-binding-domain

## Abstract

**Background:**

Data on immune responses to SARS-CoV-2 in patients with Primary Antibody Deficiencies (PAD) are limited to infected patients and to heterogeneous cohorts after immunization.

**Methods:**

Forty-one patients with Common Variable Immune Deficiencies (CVID), six patients with X-linked Agammaglobulinemia (XLA), and 28 healthy age-matched controls (HD) were analyzed for anti-Spike and anti-receptor binding domain (RBD) antibody production, generation of Spike-specific memory B-cells, and Spike-specific T-cells before vaccination and one week after the second dose of BNT162b2 vaccine.

**Results:**

The vaccine induced Spike-specific IgG and IgA antibody responses in all HD and in 20% of SARS-CoV-2 naive CVID patients. Anti-Spike IgG were detectable before vaccination in 4 out 7 CVID previously infected with SARS-CoV-2 and were boosted in six out of seven patients by the subsequent immunization raising higher levels than patients naïve to infection. While HD generated Spike-specific memory B-cells, and RBD-specific B-cells, CVID generated Spike-specific atypical B-cells, while RBD-specific B-cells were undetectable in all patients, indicating the incapability to generate this new specificity. Specific T-cell responses were evident in all HD and defective in 30% of CVID. All but one patient with XLA responded by specific T-cell only.

**Conclusion:**

In PAD patients, early atypical immune responses after BNT162b2 immunization occurred, possibly by extra-follicular or incomplete germinal center reactions. If these responses to vaccination might result in a partial protection from infection or reinfection is now unknown. Our data suggests that SARS-CoV-2 infection more effectively primes the immune response than the immunization alone, possibly suggesting the need for a third vaccine dose for patients not previously infected.

**Supplementary Information:**

The online version contains supplementary material available at 10.1007/s10875-021-01133-0.

## Introduction

The individual immune response to SARS-CoV-2 defines the COVID-19 clinical evolution, ranging from asymptomatic to mild, moderate, or severe disease with possible multi-organ failure requiring intensive care support [1].

Due to the severely impaired immune response to infection and immunization, patients with Primary Antibody Deficiencies (PAD) [2] represent a potential at-risk group in the current COVID-19 pandemic [3]. SARS-CoV-2 infected PAD patients have been reported [4–6] with a clinical presentation varying from mild symptoms to death, with many asymptomatic patients also documented. We recently showed [7] that Italian PAD patients showed a cumulative incidence and infection-fatality rate similar to the SARS-CoV-2 positive Italian general population. It is possible to consider that the low incidence might be related to the application of precautions measures our patients are used to following since PAD diagnosis. Although the infection rate and the infection-fatality rate were similar, the median age at death of PAD patients was lower compared to the general population, and most of these patients did not have predisposing comorbidities [7]. A low or even absent antibody level is generating considerable anxiety in the PAD population aware of their incapacity to mount an adequate antibody response to infection and immunization [8].

Vaccination is the safest and most effective tool to achieve a protective response in immunocompetent individuals in whom recent data demonstrated the high efficacy of SARS-CoV-2 immunization [9, 10].

The European Society for Primary Immune Deficiency (ESID) recommends that PAD patients receive SARS-CoV-2 immunization provided that vaccines are based on killed/inactivated/viruses or on the use of mRNA [11]. The rationale is, as for the influenza immunization, that immune responses may be generated despite a low or even absent antibody response [12]. We are running a study with the aim to define the short- and long-term mechanisms of impaired or preserved immune responses to SARS-CoV-2 immunization in a population of adult PAD patients.

The immune response to vaccination occurs in the germinal centers where the mechanisms of somatic mutation and affinity-selection results in the generation of high-affinity memory B-cells (MBCs) and long-lived memory plasma cells that are indispensable elements of immunological memory and exert protection in case of infection [13]. Other B-cell populations become transiently detectable in the peripheral blood. Atypical Memory B-cells (ATM) are mostly generated by extrafollicular reactions [14] where antigen selection cannot occur. Plasmablasts (PBs) are short-lived antibody producing cells found in the blood early after vaccination. Most of them will die and only some will home to the bone marrow and develop into long-lived plasma cells [15]. Thanks to the availability of fluorescent Spike protein, we have been able to determine the participation of the different cell types to the immune response in Healthy Donors (HD) and PAD patients.

We present here data on early immune responses after BNT162b2 immunization. In a cohort of immunized PAD patients, naive for SARS-CoV-2 infection or previously infected, we measured Spike-specific B- and T-cells and serum antibodies before immunization and one week after the second dose of the BNT162b2 vaccine. Results showed lack of antibody responses in the majority of patients with Common Variable Immune Deficiencies (CVID), and in all patients with X-linked Agammaglobulinemia (XLA). CVID patients generated atypical B-cell responses, as well as a variable response to the vaccination in terms of Spike-specific T-cells. XLA patients produced specific T-cell responses at the same extent of HD.

## Methods

### Study Design and Patients

We studied patients regularly followed by the Italian Care Centers for adults with primary immune deficiencies in Rome and Naples. The study was carried out in 47 patients with PAD who agreed to undergo SARS-CoV-2 immunization. Diagnosis of CVID and XLA was done according to the ESID criteria (www.ESID.com). We also included 28 immunized age-matched health care workers of the Bambino Gesù Children Hospital as healthy controls (HD). Eligible patients were informed on the study, including its safety profile and supply procedures, and signed the informed consents for vaccination and for the immunological study. The BNT162b2 vaccine was administered as prescribed, in two doses, 21 days apart. Two blood samples were obtained from each participant for serological and cellular immunity assessment at time 0 (T0), before the first dose, and seven days after the second dose (T1). During the study, patients were allowed to continue their therapies, and were monitored for their clinical status. The study was approved by the Ethical Committee of the Sapienza University of Rome (Prot. 0521/2020, July 13, 2020). The study was performed in accordance with the Good Clinical Practice guidelines, the International Conference on Harmonization guidelines, and the most recent version of the Declaration of Helsinki.

### Cell Isolation and Cryopreservation

Heparinized peripheral blood mononuclear cells (PBMCs) were isolated by Ficoll Paque™ Plus 206 (Amersham PharmaciaBiotech) density-gradient centrifugation and immediately frozen and stored in liquid nitrogen until use. The freezing medium contained 90% Fetal Bovine Serum (FBS) and 10% DMSO.

### Detection of Antigen-Specific B-Cells

To detect SARS-CoV-2 specific B-cells, biotinylated protein antigens were individually multimerized with fluorescently labeled streptavidin at 4 °C for 1 h. Recombinant biotinylated SARS-CoV-2 Spike (S1 + S2; aa16-1211) were purchased from R&D systems (BT10549). RBD were generated in-house and biotinylation was performed using EZ-LinkTM Sulfo-NHS-LC-Biotin reaction kit (ThermoScientific) following the manufacturer’s standard protocol and dialyzed overnight against PBS. Recombinant biotinylated Spike was mixed with streptavidin BUV395 (BD Bioscience) and streptavidin PE (BD Bioscience) at 25:1 ratio and 20:1 ratio, respectively. Biotinylated RBD (kindly provided by Takis) was mixed with streptavidin-FITC (BD Bioscience) at 2.5:1 ratio. Streptavidin PE-Cy7 (BD Bioscience) was used as a decoy probe to gate out SARS-CoV-2 non-specific streptavidin-binding B-cells. The antigen probes individually prepared as above were then mixed in Brilliant Buffer (BD Bioscience). 5 × 10^6^ previously frozen PBMC samples were prepared and stained with antigen probe cocktail containing 100 ng Spike per probe (total 200 ng), 27.5 ng of RBD and 20 ng of streptavidin-PE-Cy7 at 4 °C for 30 min to ensure maximal staining quality before surface staining with antibodies (listed in Supplementary materials: Antibody for staining) was performed in Brilliant Buffer at 4 °C for 30 min. MBCs were defined as CD19 + CD24 + CD27 + CD38-, ATMs were identified as CD19 + CD27-CD24-CD38-_,_ activated MBCs were gated as CD19 + CD27 + CD24-CD38-, and PBs were identified as CD19 + CD24-CD38 +  + CD27 +  + . B-cells specific for SARS-CoV-2 Spike protein were distinguished by their ability to bind biotin-labeled recombinant Spike into S + (PE single positive) or S +  + (PE-BUV395 double positive).

Stained PBMC samples were acquired on FACS LSRFortessa (BD Bioscience). At least 4 × 10^6^ cells were acquired and analyzed using FlowJo10.7.1 (BD Bioscience). Phenotype analysis of antigen-specific B-cells was performed only in subjects with at least 10 cells detected in the respective antigen-specific gate.

### Ex Vivo ELISpot Assay for IFNγ Detection

We used an IFNγ ELISpot assay (Mabtech), as described previously [16]. Briefly, isolated PBMCs were plated in duplicate, 2 × 10^5^ cells/well, stimulated with 1 µg/ml CRUDE PepMix™ SARS-CoV-2 (Spike Glycoprotein, JPT), and incubated for 24 h at 37 °C. As a positive control, PBMCs were stimulated with 5 µg/ml of phytohemagglutinin-P (PHA, Sigma). As negative control, PBMCs were plated in serum free CellGenixTM GMP (Cell Genix, GMBH). The IFN*γ* + spot-forming Unit (SFU) was counted with EliScan (Epson) by Automated ELisa-Spot Assay Video Analysis Systems (A.EL.VIS). Data were presented as the percentage of IFN*γ* SFUs obtained after pepMix stimulation, compared to the total SFUs obtained in the positive control condition (PHA).

### ELISA for Specific IgG, IgA, and IgM Detection

A semi-quantitative in vitro determination of human IgG and IgA antibodies against the SARS-CoV-2 was performed on serum samples by using the Anti-SARS-CoV-2 Spike ELISA (EUROIMMUN), according to the manufacturer’s instructions. Values were then normalized for comparison with a calibrator. Results were evaluated by calculating the ratio between the extinction of samples and the extinction of the calibrator. Results are reported as the ratio between OD sample and OD calibrator. The ratio interpretation was as follows: < 0.8 = negative, ≥ 0.8 to < 1.1 = borderline, ≥ 1.1 = positive. To detect IgM anti-RBD we developed an in-house ELISA [17]. 96-well plates (Corning) were coated for 1 h at 37 °C with 1 μg/mL of purified SARS-CoV-2 RBD protein (Sino Biological). After washing with PBS 1 × /0.05% Tween and blocking with PBS 1 × /1% BSA, plates were incubated for 1 h at 37 °C with diluted sera (1:100). After washing again, plates were incubated for 1 h at 37 °C with peroxidase-conjugated goat anti-human IgM antibody (Jacksons ImmunoResearch Laboratories). The assay was developed with o-phenylenediamine tablets (Sigma-Aldrich) as a chromogen substrate. Absorbance at 450 nm was measured, and IgM concentrations were calculated by interpolation from the standard curve based on serial dilutions of monoclonal human IgM antibody against SARS-CoV-2 Spike-RBD (Invivogen).

### Statistical Analysis

Data from CVID and XLA patients have been separately analyzed and compared with data of age-matched controls (HD). Demographics were summarized with descriptive statistics (median and IQR for continuous values). Immunological, and clinical variables were compared between the different study times. A univariate analysis assessed the impact of variables of interest. Values were compared by the non-parametric Kruskal–Wallis test and, if not significant, the Wilcoxon matched pair signed-rank test or the two-tailed Mann–Whitney U-test were used. Differences were deemed significant when *P* < 0.05. Statistical Package for Social Sciences version 15 (SPSS Inc., 233 South Wacker Drive, 11th Floor, Chicago) has been used for the analysis.

## Results

### Patients

In the study on Spike-specific antibody responses, we included 41 patients with CVID, 6 patients with XLA, and 28 HD who received two doses of BNT162b2 vaccine. Demographic, clinical, and immunological data at baseline of all 47 patients enrolled and individual data are shown in Table S1 and Table S2. Immunoglobulin replacement treatment was continued in all PAD patients.

Thirty-four CVID patients did not experience a previous SARS-CoV-2 infection, as shown by negativity at the periodical nasopharyngeal swab by PCR testing done every time a patient is attending a hospital site and in all patients with family contacts [7]. Seven CVID patients were vaccinated with two doses of BNT162b2 vaccine at least 3 months after recovering from a mild COVID-19. None of the PAD patients was infected with SARS-CoV-2 after completing the two doses vaccination cycle, with the exception of one CVID patient who was infected two months after completing the vaccination, when his Spike-specific IgG level was 2.5 OD ratio. He was treated with monoclonal antibodies within 24 h from the onset of a mild fever and since then he did not show any additional COVID-19 symptoms. Twenty-six CVID, 6 XLA patients, and 28 HD were included in the study of the specific B- and T-cell responses. We did not analyze specific responses in 15 CVID patients due to the lack of samples.

### SARS-CoV-2 Antibodies

#### Common Variable Immunodeficiency

**Fig. 1:**
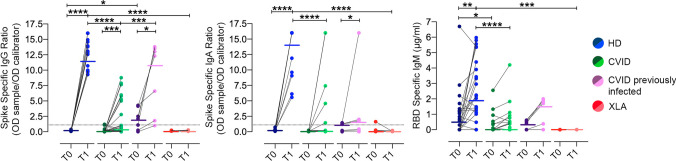
RBD-specific IgM and Spike-specific IgG and IgA antibodies in HD (blue circles), CVID patients (green circles), CVID previously infected patients (pink circles) and XLA (red circles), before (T0, dark color circles) and one week after the second dose (T1, lighter color circles) of BNT162b2 vaccine. For each group the median is shown as a bar. * *P* ≤ 0.05, ** *P* ≤  0.01, *** *P* ≤  0.001, **** *P* < 0.0001. Positive cut-off value is represented by a dashed line. *N* = 28 HD, *N* = 34 CVID, *N* = 6 XLA patients, and *N* = 7 CVID previously SARS-CoV-2 infected

CVID is the most prevalent symptomatic PAD [14] with reduced or absent antibody response to infections and immunization, paucity of switched memory B-cells and dysregulated T-cell responses. Spike-specific IgG and IgA, and RBD-specific IgM antibodies were evaluated at T0 and T1. Antibodies to SARS-CoV-2 antigens in 41 CVID patients and in 28 HD are shown in Fig. 1 and Table 1. In all HD, anti-Spike IgG and IgA significantly increased in post-immunization samples, while anti-RBD antibodies of IgM isotype were already detectable at T0, reflecting the presence of natural or cross-reactive antibodies [18]. IgM increased at T1, with a wide variability between HD (Fig. 1).

**Table 1:** Summary of antibody responses and specific B-cells in CVID and XLA patients

	HD *n* = 28		HD T0 vs T1	CVID *n* = 41		CVID T0 vs T1	CVID vs HD (T1)	XLA *n* = 6		XLA T0 vs T1	XLA vs HD (T1)
	Median	IQR (25th–75th)	*P* value	Median	IQR (25th–75th)	*P* value	*P* value	Median	IQR (25th–75th)	*P* value	*P* value
SARS-Cov-2 antibodies											
IgG OD ratio, T0	0.10	0.00–0.15	< 0.0001	0.12	0.07–0.24	< 0.0001	< 0.0001	0.15	0.11–0.16	> 0.999	< 0.0001
IgG OD ratio, T1	13.00	11-.00–12.00		0.39	0.13–5.25			0.12	0.10–0.19		
IgM µg/mL, T0	0.55	0–1.24	0.0014	0	0–0.42	0.052	< 0.0001	0.00	–	–	0.004
IgM µg/mL, T1	2.20	1.13–4.40		0.14	0.00–0.92			0.00	–		
IgA OD ratio, T0	0	0.2–0.3	*P* < 0.0001	0.05	0.04–0.11	0.070	< 0.0001	0.13	0.05–0.22	0.562	< 0.0001
IgA OD ratio, T1	9	11–13		0.07	0.04–0.20			0.05	0.04–0.14		

	HD* n* = 28		HD T0 vs T1	CVID* n* = 26		CVID T0 vs T1	CVID vs HD (T1)	XLA* n* = 6		XLA T0 vs T1	XLA vs HD (T1)
	Median	IQR (25th–75th)	*P* value	Median	IQR (25th–75th)	*P* value	*P* value	Median	IQR (25th–75th)	*P* value	*P* value
Spike-specific cells											
MBC S + %, T0	0.21	0.13–0.33	0.001	0.05	0–0.15	0.19	< 0.0001	0	0–0	–	< 0.0001
MBC S + %, T1	0.44	0.21–0.65		0.08	0–0.2			0	0–0	–	
MBC S + + %, T0	0	0–0.002	< 0.0001	0	0–0	0.06	< 0.0001	0	0–0	–	0.0004
MBC S + + %, T1	0.016	0.005–0.04		0	0–0.002			0	0–0	–	
Activated MBC S + T0	0	0–0	0.06	0	0–0	–	< 0.0001	0	0–0	–	< 0.0001
Activated MBC S + T1	0	0–0.46		0	0–0			0	0–0	–	
Activated MBC S + + T0	0.0	0–0	0.030	0	0–0	–	< 0.0001	0	0–0	–	< 0.0001
Activated MBC S + + T1	0.21	0–0.65		0	0–0			0	0–0	–	
ATM S + %, T0	0	0–0	0.0001	0.0	0–0	0.019	0.43	0	0–0	–	0.08
ATM S + %, T1	0.06	0–0.35		0.0	0–0.31			0	0–0	–	
AMT S + + %, T0	0	0–0	0.250	0	0–0	> 0.999	0.4	0	0–0	–	> 0.999
ATM S + + %, T1	0	0–0		0	0–0			0	0–0	–	
PB S + %, T0	0	0–0	< 0.0001	0	0–0	0.001	0.027	0	0–0	> 0.999	0.0006
PB S + %, T1	1.61	0.67–2.7		0.72	0–1.81			0	0–0.25		
PB S + + %, T0	0	0–0	< 0.0001	0	0–0	0.125	0.005	0	0–0	> 0.999	0.17
PB S + + %, T1	0.15	0–0.51		0	0–0			0	0–0.25		
RBD + %, T0	1	0–3	< 0.0001	0–0	0–0	–	< 0.0001	0–0	0–0	–	< 0.0001
RBD + %, T1	16	4–39.75		0–0	0–0	–		0–0	0–0	–	
Spike-specific T-cells (IFN-g SFU × 10^6^ cells) T0	2.5	0–68.7	0.041	0	0–9	0.151	0.001	37.5	0–50.5	0.041	0.937
Spike-specific T-cells (IFN-g SFU × 10^6^ cells) T1	115	70–250		7	0–35			152.5	41.2–185		

Among 34 CVID patients not previously infected by SARS-CoV-2, we observed an interindividual variability in the production of anti-Spike IgG and IgA (Fig. 1). In more detail, 7/34 patients (20.6%) developed both anti-Spike IgG and IgA antibodies (≥ 1.1 OD ratio), and one patient responded with IgG only. However, the level of antibodies in the few patients who produced specific IgG and IgA was significantly lower than the level measured in vaccinated HD (T1: CVID IgG median 5.88 OD ratio IQR 5.06–7.83 vs HD 13.10 OD ratio IQR 9.98–16.00; *P* < 0.0001; IgA CVID median 6.00 OD ratio (IQR 1.43–16.00) vs HD 16.00 OD ratio (IQR 16.00–16.00) *P* = 0.006). Specific S1 IgA were related to total serum IgA levels (R 0.48, *P* < 0.0001). After vaccination, anti-RBD IgM did not increase in CVID (T0: 0.00 µg/mL (IQR 0–0.37) to T1: 0.00 µg/mL (IQR 0.00–0.52), *P* = 0.061, Fig. 1).

IgG were already detectable at T0 (median 2.10 OD ratio (IQR 0.25–4.16) in 4/7 CVID patients who were previously infected with SARS-CoV-2 (3–8 months before vaccination), suggesting that IgG might persist after primary infection in some patients. In 6/7 patients, IgG increased at T1 (median 12.31 OD ratio (IQR 1.80–13.50), showing that IgG were boosted by the subsequent immunization with two doses of BNT162b2 vaccine (Fig. 1). To note, CVID patients who were previously infected raised higher anti-Spike IgG levels at T1 than patients naïve to SARS-CoV2 infection (*P* = 0.0002).

#### X-linked Agammaglobulinemia

As expected, due to the lack of B-cells and the consequent lack of serum antibody in XLA [19], anti-Spike- and anti-RBD-specific antibodies were not generated after immunization (Fig. 1).

### Spike SARS-CoV-2 Memory B-Cells

**Fig. 2:**
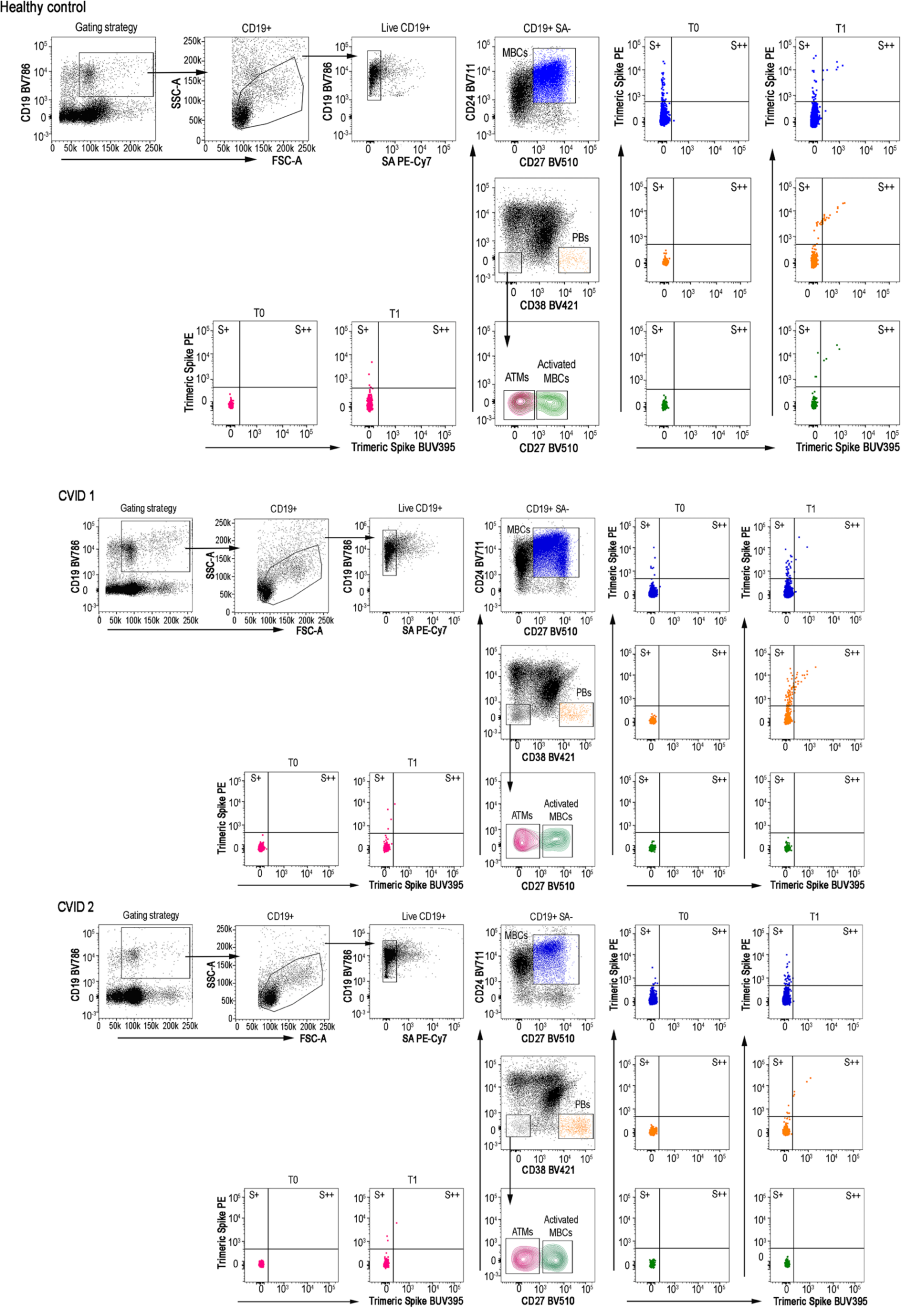
Gating strategy to identify S + and S +  + MBCs, PBs, ATM , and activated MBCs. One HD and two CVID subjects are shown. We analyzed CD19 + B-cells, included in the live gate. SA PE-Cy7 was used as a decoy probe to gate out streptavidin-binding B-cells from further analysis. MBCs were identified as CD24 + CD27 + CD38-; PBs as CD24-CD27 +  + CD38 +  + ; ATMs as CD24-CD27-CD38-CD21- -; and activated MBCs as CD24- CD38-CD21-CD27 + . Flow cytometry plots show the staining patterns of SARS-CoV-2 antigen probes in the indicated B-cell populations during the follow-up. S + are B-cells that are Spike-PE + , but Spike-BUV395-. S +  + are instead Spike-PE + and SpikeBUV395 + . The color code identifies Spike + and +  + in MBCs (blue), PBs (orange), ATMs (dark red) ), and Activated MBCs (green)

**Fig. 3:**
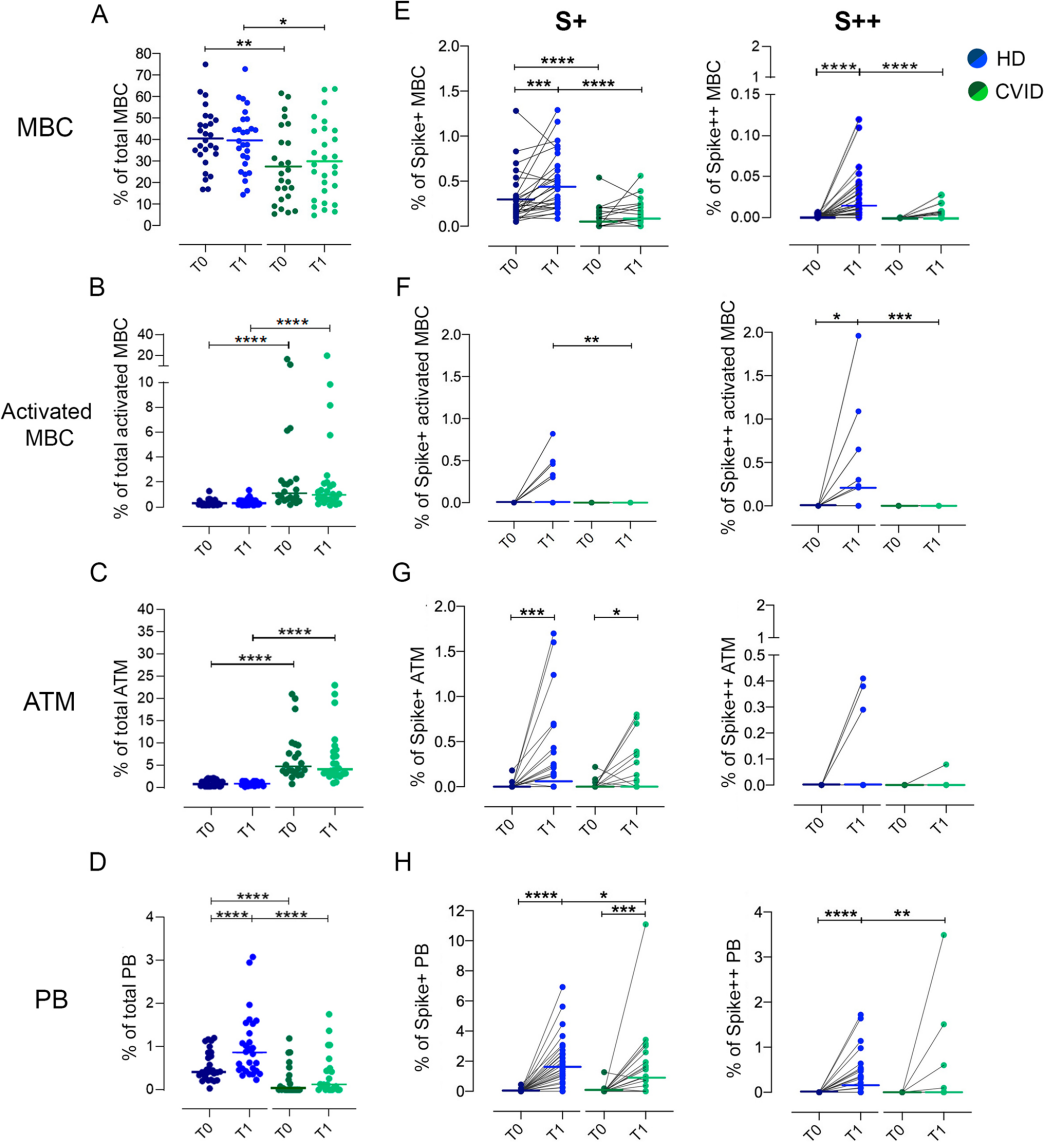
Peripheral blood B-cells subsets, low and high binding capacity B-cells for recombinant Spike protein in HD (blue circles) and in CVID (green circles) before (T0, dark color circles) and after two doses of the BNT162b2 vaccine (T1, lighter color circles). We show the frequencies of peripheral blood MBCs (panel **a**), Activated MBCs (**b**), ATMs (**c**), and PBs (**e**) in HD (blue circles) and CVID (green circles). The frequency of S + and S +  +  B-cells inside each identified B-cell population is shown (**e**, **f**, **g**, and **h**). Medians are plotted as horizontal bars and statistical significance were determined using two-tailed Mann–Whitney U-test or Wilcoxon matched-pairs signed-rank test. *P < 0.05, **P < 0.01, ***P < 0.001; ****P < 0.0001. *N* = 28 HD and *N* = 26 CVID patients. B-cells subsets were defined as following: MBCs CD19 + CD24 + CD27 + CD38-; activated MBCs CD19 + , CD27 + CD24-CD38-; specific ATMs CD19 + CD27-CD24-CD28-; PBs CD19 + CD24-CD38 +  + CD27 +  +

High specificity and affinity are the most important characteristics of protective MBCs, generated by the adaptive immune system in response to infection or vaccination [20]. MBCs, ATM, activated MBCs, and PBs were identified by flow-cytometry, based on the expression of CD19, CD27, CD24, and CD38 markers (Fig. 2, gating strategy). MBCs were identified as CD19 + CD24 + CD27 + CD38- cells; ATMs were identified as CD19 + CD27-CD24-CD38- cells, which are also CD21 negative [21, 22]. Activated MBCs also lack CD21, but express CD27 [23, 24]. PBs were identified as CD19 + CD24-CD38 +  + CD27 +  + cells. Overall, CVID patients showed lower frequency of MBCs and PBs, and higher frequency of ATMs than HD at all study times (Fig. 3, and Table 1). This baseline pattern that distinguishes the B-cell populations of HD from those of CVID patients affects the specific response to vaccination.

B-cells specific for SARS-CoV-2 Spike protein were distinguished by their ability to bind biotin-labeled Spike protein. Thanks to the availability of biotin-labeled Spike protein coupled to an extremely high brightness fluorescence dye (PE) and the same biotin-labeled Spike protein coupled to a moderate brightness fluorescent dye (BUV395) [25] (Fig. 2, gating strategy) we were able to distinguish MBCs with low (PE single positive, S +) or high binding capacity (PE-BUV395 double positive, S + +) for Spike protein. RBD-specific B-cells were identified by RBD-biotin labeled with streptavidin-FITC (Fig. S1).

All HD responded to immunization by generating a classical B-cell response with S + and S +  + MBCs (*P* = 0.001 and *P* < 0.0001, respectively) and S +  + activated MBCs (*P* = 0.030). HD generated S + ATMs (*P* = 0.0001), and S + and S +  + PBs (*P* < 0.0001) (Fig. 3 and Table 1), demonstrating that these populations are induced by the immune response to vaccination [26]. The progression from single to double positive memory B-cells is clearly demonstrated in immunized HD. MBCs only positive for PE (S +) are mostly of IgM isotype and present before immunization, as expected by low-affinity innate/cross-reactive memory B-cells. In contrast, S +  + MBCs appear only after immunization and are of switched isotype (IgM-) because they are the product of the germinal center reaction where affinity maturation and class-switching has occurred (Fig. S2).

On the contrary, CVID patients did not generate classical and activated MBCs, but vaccination induced S + ATMs B-cells only (*P* = 0.019) (Fig. 3 and Table 1). This was observed only in about one third of CVID patients, where the frequency of S + ATMs at least doubled seven days after the second dose of vaccine. ATMs originally observed in tonsils and later in peripheral blood, are observed in conditions of chronic antigen stimulation [27] and may be produced by extra-follicular reactions or failed and incomplete germinal center reactions [28]. As expected, S +  + PBs were produced in HD at T1, but not in CVID, except for three patients who also produced specific IgG and IgA. About one third of CVID patients were able to generate S + PBs (*P* = 0.001) that are possibly short-lived PBs derived from the non-canonical pathway of ATMs.

**Fig. 4:**
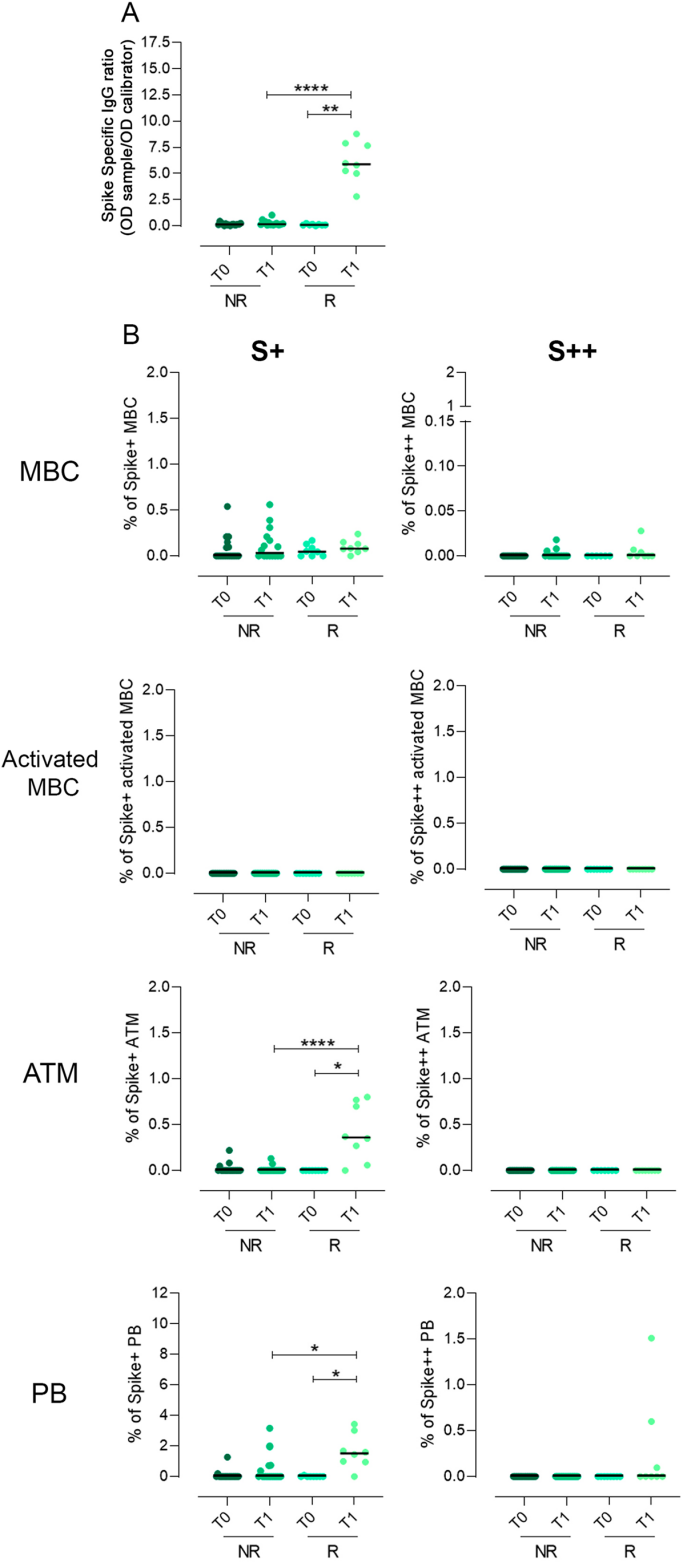
Spike-specific IgG, low and high binding capacity B-cells for recombinant Spike protein in CVID who did not develop Spike-specific IgG (NR) and in CVID who developed Spike-specific IgG (R) before (T0) and after two doses of the BNT162b2 vaccine (T1). **a** Dot plot depicts the levels of Spike-specific IgG. **b** The frequency of S + and S +  +  B-cells inside each identified B-cell population, in R and NR CVID patients, is shown. Medians are plotted as horizontal bars and statistical significance were determined using two-tailed Mann–Whitney *U*-test or Wilcoxon matched-pairs signed-rank test. *P < 0.05, **P < 0.01, ****P < 0.0001. *N* = 16 NR and *N* = 8 R

In conclusion, the response to vaccination was not homogeneous among CVID patients: 8 of 34 patients developed anti-Spike IgG after immunization. These responder patients (R) had significantly higher levels of low-affinity S + ATMs and S + PBs in comparison to patients who did not respond (NR) (*P*  < 0.0001 and *P*  = 0.0034, respectively). None of the CVID responder patients generated specific MBCs or PBs (Fig. 4).

Among total Spike positive MBCs (S + plus S + +), we also identified RBD-specific cells. RBD + cells represent a minority of the MBCs generated by vaccination and significantly increased at T1 in HD (*P*  < 0.0001) (Table 1 and Fig. S1). These cells with high specificity for the RBD of SARS-CoV-2 producing most of the neutralizing antibodies are undetectable in CVID patients indicating the incapability of CVID B-cells to undergo somatic mutation in the germinal center and thus to generate this new specificity.

XLA patients lack B-cells and consequently did not generate any specific B-cells (Table 1).

### Spike SARS-CoV-2 T- Cells

In HD, vaccination induced the generation of Spike-specific T-cells evaluated as Interferon gamma (IFN-*ɣ*) Colony Forming Units that significantly increased (from T0: median 2.5 SFU (IQR 0–0.69) to T1: 115 median SFU (IQR 0.13–5.25), *P* = 0.041). Differently from what observed after influenza vaccination [29], in CVID patients, Spike-specific T-cells producing IFN-ɣ did not increase (from T0: median 0 SFU (IQR 0–9) to T1: median 7 SFU (IQR 0–35), *P* = 0.151) and were significantly lower than in HD (*P* = 0.0001). Nevertheless, XLA patients developed a significant T-cell response to vaccination. Indeed, Spike-specific T-cells were detected in 5 out of 6 tested patients (from T0: median 37.5 SFU (IQR 0–50.5) to T1: median 152.5 SFU (IQR 41–185), p = 0.041), with a negligible difference compared to those one established in HD (*P* = 0.937).

## Discussion

Effective vaccines against SARS-CoV-2 are being administered worldwide with the aim of terminating the COVID-19 pandemic. As for all immunizations, the efficacy has been linked to the production of specific antibodies, which increase in response to all vaccines in use [30, 31]. The majority of patients with PAD show clinical and immunological characteristics implicating a functional impairment of the B-cell compartment, and a dysregulation of T-cell responses causing hypo-gammaglobulinemia or agammaglobulinemia and susceptibility to a wide range of microbial infections [32]. Despite the severely impaired antibody responses, when infected with SARS-CoV-2, one fourth of adult PAD patients remained asymptomatic and half of them showed a mild disease [7]. It should be considered that a protective effect against severe COVID-19 could be due to the immune-modulatory effects on innate immunity exerted by immunoglobulin therapy also when administered at replacement dosages [33]. However, data on immunogenicity of SARS-CoV-2 vaccine in patients with Inborn Errors of Immunity are few and limited to anecdotal cases or heterogeneous cohorts [34]. After infection, a robust T-cells activity and humoral immunity against SARS-CoV-2 structural proteins in some patients with antibody deficiency has been described in five patients [35]*.* Consistent with the finding of a good antibody response after infection, also immunization with an mRNA COVID-19 vaccine resulted in high-level antibody titers in 11 patients with immune deficiency [36] and in patients who were infected before immunization [37].

Although the natural course of COVID-19 is primarily characterized by the function of the innate immune system, with a secondary involvement of T- and B-cells, vaccines are designed to force the adaptive immune system to generate neutralizing antibodies and memory B- and T-cells that effectively protect from COVID-19.

Here we showed that while HD produced specific antibodies and generated MBCs and activated MBCs with high binding capacity that significantly increased after immunization, these responses are lacking in all XLA and severely impaired in CVID patients after SARS-CoV-2 immunization, suggesting an incomplete response. Moreover, the few CVID patients who responded to immunization by anti-Spike IgG also developed ATMs and PBs with low binding capacity for Spike, instead of a response by MBCs. These responses are probably short-lived and should be reassessed over time. Interestingly, in one third of CVID patients vaccination induced B-cells specific for recombinant Spike protein inside the ATM population, possibly suggesting that the B-cell responses occurred mostly at extra-follicular sites [38], as recently demonstrated [39]. In line with this hypothesis, RBD + B-cells were undetectable in CVID patients, whereas RBD + B-cells represent 20% of the specific anti-Spike response in HD (unpublished data) able to develop and successfully terminate the germinal center reaction [40]. Thus, CVID patients were able to respond to immunization by two doses of BNT162b2 with atypical lineage B-cells induced by a primary exposure to a novel antigen. It has been suggested that atypical B-cells are short-lived activated cells, in the process of differentiating into plasma cells [26, 41]. In addition, interesting information was gained by the parallel study of the T-cell responses, showing a robust generation of Spike-specific T-cells in all but one patient with XLA, and in HD. Specific-T-cell responses were induced in a minority of CVID patients with a variable frequency. Based on data on response to influenza virus immunization [11], we expected a more efficient generation of specific T-cells. However, this was not the case. While after influenza virus immunization T-cells are generated after multiple exposures to viral antigen following infection and immunization, SARS-CoV-2 is a pathogen never encountered before, since SARS-CoV-2 Spike and the RBD domains are district from the S proteins of most members of the family of coronavirus [42]. Then, it is possible that the first antigenic stimulation was not sufficient to induce an early T-cell response.

Based on these data, how can we explain the paucity of symptoms or the mild COVID-19 course in PAD patients, and what might we expect after immunization?

To pathogens for which there is no preexisting immunity, our organism reacts by rapidly engaging the innate immune system with the intent to limit the infection. The adaptive immune response develops slowly and needs two weeks to generate the most specific and effective defensive tools. However, the vast majority of PAD patients infected with SARS-CoV-2 did not show signs of hyper-activation of the innate immunity [3–5]. This could possibly be due to the immunomodulatory effects on innate immune cells of replacement with polyvalent immunoglobulins [33], and to a poor adaptive immunity response. This pattern of immune responses resembles what we have already shown in asymptomatic immunocompetent subjects [43], and further demonstrated that a balanced cytokine production resulting from a functional but not hyper-reactive innate immunity and a poor adaptive immunity are the conditions associated with an early benign COVID-19 course. Our data are partially in contrast to observations reported in small cohorts of PAD, showing that the majority of CVID patients are able to respond to the BNT162b2 vaccine [34]. Differently from previous studies, we described a homogeneous cohort of patients, separately analyzing CVID and XLA at different time points.

In summary, a minority of PAD patients showed adaptive, atypical immune responses after SARS-CoV-2 immunization. If these responses to vaccination might result in a partial protection from infection or reinfection is now unknown, since we do not know the levels of antibodies or the frequency of specific B- and T-cells required to protect from the infection.

It should be remembered here that each PAD patient should be studied as unique in terms of cellular and humoral responses due to the variability of their underlying immune deficiency. In our series, antibody response after two doses of BNT162b2 immunization—overlapping that of HD—was found in one patient homozygous for TNFRSF13B mutation, but not in two patients with a heterozygous TNFRSF13B mutation. In a previous study, we demonstrated that CVID patients with biallelic TNFRSF13B mutations responded also to polysaccharide vaccines, while CVID with only one TNFRSF13B mutation showed an impaired response to vaccination [44].

A major limitation of our study is the short time of observation after vaccination. We do not know if the antibody and cellular responses might persist or decline over time, nor if PAD patients might show delayed responses. However, we do not expect to observe major changes in the immune response to SARS-CoV-2 with time after immunization. Is it possible to hypothesize a boost of specific B-cell immunity? In those CVID patients who were previously infected with SARS-CoV-2, IgG were detectable when they received two vaccine doses administered at least three months after SARS-CoV-2 infection recovery, suggesting that if IgG were produced, they might persist after the primary infection. Moreover, in these previously infected CVID patients, IgG response was boosted by the subsequent immunization. After immunization, anti-Spike IgG were higher than in patients who were not previously infected, showing that SARS-CoV-2 infection more effectively primed the immune response than the vaccine alone. Whether it may be useful to administer a third vaccine dose to CVID patients not previously infected, should be demonstrated as suggested for patients with solid organ transplantation undergoing immunosuppressive treatment [45].

Moreover, data available from T-cell immunity after influenza virus vaccination in PAD [11] might suggest a possible strategy aimed to boost also the SARS-CoV-2 T-cell specific responses by additional vaccine doses.

Since antibody titers are not a precise indicator of the magnitude of memory cells [46] our strategy is to follow-up our cohort by serological and cellular investigations. The only epidemiological observation we have for now is that one CVID patient experienced an infection by SARS-CoV-2 three months after completing the two doses vaccination. He remained asymptomatic possibly due to the prompt administration of monoclonal antibodies [47]. For now, SARS-CoV-2 positive CVID patients might benefit from these new treatments. Prevention of infection may be achieved by the presence of SARS-CoV-2 antibodies in the coming lots of gamma globulins, regularly used to substitute the missing or partial response to infections and vaccination.

## Supplementary Information

Below is the link to the electronic supplementary material.

Supplementary file1 (DOCX 853 kb)

Supplementary file2 (JPG 413 kb)

Supplementary file3 (JPG 398 kb)

## Data Availability

The authors confirm that the data supporting the findings of this study are available within the article and its supplementary materials.
