# Supplementary material for: SARS-CoV-2 Vaccine Induced Atypical Immune Responses in Antibody Defects: Everybody Does their Best
Source: J Clin Immunol. 2021 Oct 20;41(8):1709–22. doi: 10.1007/s10875-021-01133-0 (PMC8527979; doi:10.1007/s10875-021-01133-0)

**Supplementary data**

**Antibodies for staining**

|  | **Clone** | **Catalog number** |
| --- | --- | --- |
| CD19 BV786 | SJ25C1 | 563325 |
| CD24 BV711 | ML5 | 563401 |
| CD27 BV510 | T-271 | 740167 |
| CD38 BV421 | HIT2 | 562444 |
| IgG BV650 | G18-145 | 740596 |
| IgM APC | Polyclonal | 709-136-073 |
| Streptavidin PE |  | 554061 |
| Streptavidin BUV395 |  | 564176 |
| Streptavidin FITC |  | 554060 |
| Streptavidin PE-Cy7 |  | 557598 |

**Supplementary Tables**

**Supplementary Table 1. Demographic, clinical and immunological characteristics of immunized CVID, XLA patients, and HD**

|  | Age  (years) | Sex  (F/M) | Age at diagnosis (years) | IgG  at diagnosis  (mg/dL) | IgG  at T0  (mg/dL) | IgM  at T0  (mg/dL) | IgA  at T0  (mg/dL) | CLD | Autoimmunity | LRTI,  rate  per year | Lymphocyte  (cell/mm3) | CD19+  (cell/mm3) | CD27+  IgM-IgD-  (cell/mm3) | CD21low  (cell/mm3) | CD4+  (cell/mm3) |
| --- | --- | --- | --- | --- | --- | --- | --- | --- | --- | --- | --- | --- | --- | --- | --- |
| CVID  (n=41) | 49  (42-59) | 9/32 | 36  (60-44) | 320  (280-380) | 700  (620-800) | 23  (7-30) | 15  (5-36) | 9/41 | 18/41 | 1  (0-3) | 1500  (1010-2080) | 146,5  (22.7 – 249.2) | 3  (1-10.7) | 5.5  (1-48.5) | 540  (423-873) |
| XLA  (n=6) | 41  (28.7-51.7) | 0/6 | 23  (5.7-29.7) | 0  (0-22.5) | 765  (725.5-780) | 0  (0-1.5) | 0  (0-2.2) | 3/6 | 0/6 | 1  (0.2-1.7) | 1975  (1855-2612) | 0  (0-0) | 0  (0-0) | 0  (0-0) | 499  (424-610) |
| HD  (n=28) | 41.7  (34.4-58.8) | 17/11 | Nap | Nap | 1105  (604-1909) | 136  (61-301) | 132  (59-297) | Nap | Nap | Nap | 1600  (1300-1900) | 210  (160-270) | 24  (4-13) | 9  (2-5) | 700  (600-980) |

XLA: X-linked Agammaglobulinemia; CVID: Common Variable Immune Deficiency; M: male; F: Female; CLD: Chronic Lung Disease; LRTI Low Respiratory Tract Infections. Nap: not applicable

**Supplementary Table 2. Individual characteristics at the enrollment of 41 CVID and 6 XLA included in the analysis.**

| **ID** | **Diagnosis** | **Age (years)** | **Sex** | **Age at diagnosis (years)** | **IgG at diagnosis (mg/dL)** | **IgG at T0**  **(mg/dL)** | **IgM at T0**  **(mg/dL)** | **IgA at T0 (mg/dL)** | **CLD** | **Autoimmunity** | **Immuno-suppressive treatment** | **LRTI, rate**  **per year (2019)** | **Lymphocyte count**  **(cells/mm3)** | **CD19+**  **(cell/mm3)** | **CD27+ IgM-IgD-**  **(cell/mm3)** | **CD21low (cell/mm3)** | **CD4+**  **(cell/mm3)** |
| --- | --- | --- | --- | --- | --- | --- | --- | --- | --- | --- | --- | --- | --- | --- | --- | --- | --- |
| 1 | CVID | 45 | F | 43 | 320 | 750 | 68 | 60 | No | No | No | 0 | 2260 | 450 | ND | ND | 1400 |
| 2 | CVID | 56 | F | 37 | 280 | 680 | 2 | 0 | No | No | No | 4 | 1700 | 113 | 0 | 18 | 452 |
| 3 | CVID | 78 | F | 66 | 134 | 800 | 13 | 34 | COPD | No | No | 0 | 2200 | 146 | 6 | 67 | 728 |
| 4 | CVID | 82 | F | 43 | 350 | 690 | 158 | 7 | COPD | Yes, CD, alopecia | No | 5 | 2100 | 336 | 17 | 144 | 441 |
| 5 | CVID | 60 | M | 52 | 320 | 701 | 7 | 23 | No | No | No | 0 | 460 | 16 | 0 | 1 | **260** |
| 6 | CVID | 74 | M | 35 | 136 | 700 | 0 | 0 | COPD | Yes, Vitiligo | No | 5 | 1340 | 294 | 0 | 182 | 519 |
| 7 | CVID | 47 | F | 34 | 301 | 820 | 11 | 0 | No | Yes, ITP/AHE | Rituximab, Prednisone | 0 | 1080 | 23 | 1 | 10 | 812 |
| 8 | CVID | 46 | F | 34 | 28 | 880 | 3 | 13 | No | Yes, Thyroiditis | No | 0 | 1180 | 71 | 1 | 1 | 423 |

| 9 | CVID | 65 | F | 38 | 375 | 990 | 0 | 0 | ILD | Yes, RA | Tocilizumab  Metilprednisonen,  Rituximab | 1 | 720 | 152 | 2 | 46 | 480 |
| --- | --- | --- | --- | --- | --- | --- | --- | --- | --- | --- | --- | --- | --- | --- | --- | --- | --- |
| 10 | CVID | 62 | F | 48 | 312 | 670 | 87 | 60 | No | No | No | 0 | 1070 | 91 | 19 | 5 | 976 |
| 11 | CVID | 47 | F | 33 | 396 | 510 | 2 | 3 | No | No | No | 1 | 610 | 14 | 1 | 5 | **367** |
| 12 | CVID | 54 | F | 44 | 144 | 480 | 50 | 11 | No | Yes, ITP | Prednisone | 0 | 4500 | 184 | 9 | 86 | 444 |
| 13 | CVID | 32 | F | 19 | 280 | 620 | 0 | 0 | No | Yes, CD | No | 0 | 3290 | 349 | 0 | 56 | 1222 |
| 14 | CVID | 48 | F | 43 | 105 | 520 | 25 | 50 | No | No | No | 1 | 3160 | 287 | 7 | ND | 1820 |
| 15 | CVID | 44 | M | 34 | 269 | 610 | 15 | 24 | No | No | No | 0 | 970 | 161 | 2 | ND | 540 |
| 16 | CVID | 59 | F | 31 | 340 | 480 | 50 | 25 | COPD, lobectomy | Yes, IBD | Ustekimumab,  metilprednisone | 4 | 850 | 0 | 0 | 0 | 411 |
| 17 | CVID | 22 | F | 13 | 300 | 755 | 15 | 5 | No | Yes, ITP/AHE | Prednisone Rituximab | 0 | 1003 | 250 | 10 | ND | 528 |
| 18 | CVID | 59 | F | 59 | 350 | 620 | 29 | 21 | No | No | No | 1 | 1536 | 485 | 13 | ND | 436 |
| 19 | CVID | 55 | M | 36 | 380 | 900 | 7 | 5 | No | Yes, ITP | Prednisone | 2 | 1070 | 22 | 2 | 2 | **308** |
| 20 | CVID | 29 | M | 17 | 355 | 580 | 7 | 36 | No | No | No | 0 | 1970 | 177 | ND | ND | 720 |
| 21 | CVID | 80 | M | 71 | 313 | 580 | 59 | 50 | COPD | No | No | 1 | 1290 | 193 | 5 | ND | 645 |
| 22 | CVID | 31 | M | 16 | 400 | 650 | 25 | 24 | No | Yes, IBD | Prednisone | 0 | 1500 | 70 | 3 | 6 | **299** |
| 23 | CVID | 71 | F | 70 | 380 | 620 | 25 | 52 | No | No | No | 0 | 2020 | 220 | 40 | ND | 920 |
| 24 | CVID | 56 | M | 36 | 360 | 580 | 25 | 50 | No | No | No | 4 | 1900 | 152 | 19 | ND | 760 |
| 25 | CVID | 66 | F | 59 | 412 | 890 | 55 | 70 | No | Yes, Thyroiditis | No | 5 | 2100 | 147 | 84 | ND | 1050 |
| 26 | CVID | 59 | F | 38 | 350 | 710 | 28 | 52 | No | Yes, Thyroiditis | No | 5 | 2390 | 585 | 88 | 18 | 1892 |
| 27 | CVID | 56 | M | 49 | 253 | 670 | 29 | 35 | COPD | Yes, ITP | No | 5 | 840 | 34 | 2 | ND | 352 |

| 28 | CVID | 27 | F | 21 | 310 | 720 | 0 | 0 | No | No | No | 2 | 2080 | 249 | 7.4 | 4 | 873 |
| --- | --- | --- | --- | --- | --- | --- | --- | --- | --- | --- | --- | --- | --- | --- | --- | --- | --- |
| 29 | CVID | 46 | M | 47 | 305 | 800 | 9 | 8 | COPD | Yes, ITP | No | 2 | 880 | 0 | 0 | 0 | 404 |
| 30 | CVID | 23 | M | 30 | 387 | 670 | 30 | 6 | No | Yes, ITP | No | 2 | 2970 | 178 | 5 | 0 | 1217 |
| 31 | CVID | 55 | F | 36 | 280 | 740 | 10 | 15 | No | No | No | 3 | 1160 | 0 | 3 | 0 | 278 |
| 32 | CVID | 24 | F | 15 | 280 | 810 | 20 | 0 | No | Yes, ITP | Mycophenolate  Prednisone | 1 | 550 | 5.5 | 0.3 | 1.7 | 373 |
| 33 | CVID | 30 | F | 25 | 202 | 740 | 171 | 6 | No | No | No | 3 | 2300 | 20 | 5 | ND | 540 |
| 34 | CVID | 36 | F | 23 | 387 | 560 | 8 | 15 | No | Yes, ITP | Prednisone | 4 | 1010 | 20 | 3 | ND | 393 |
| 35 | CVID | 72 | M | 36 | 380 | 670 | 59 | 18 | COPD | No | No | 3 | 1260 | 38 | 1.1 | 6.4 | 441 |
| 36 | CVID | 48 | M | 43 | 450 | 700 | 17 | 45 | No | No | No | 1 | 1340 | 27 | 19 | 0.8 | 549 |
| 37 | CVID | 42 | F | 33 | 432 | 890 | 7 | 25 | No | Yes, ITP | Prednisone | 1 | 610 | 18 | ND | ND | 427 |
| 38 | CVID | 27 | M | 19 | 400 | 780 | 30 | 40 | No | No | No | 1 | 2080 | 250 | 7.5 | 4.2 | 873 |
| 39 | CVID | 49 | M | 33 | 412 | 670 | 25 | 6 | No | No | No | 1 | 1990 | 340 | 0 | ND | 1114 |
| 40 | CVID | 59 | F | 55 | 208 | 890 | 50 | 12 | No | No | No | 0 | 2010 | 120 | ND | ND | 924 |
| 41 | CVID | 45 | F | 30 | 358 | 910 | 23 | 5 | No | No | No | 0 | 1590 | 16 | ND | ND | 826 |
| 42 | XLA | 27 | M | 1 | 0 | 750 | 0 | 0 | No | No | No | 2 | 1840 | 0 | 0 | 0 | 415 |
| 43 | XLA | 64 | M | 29 | 0 | 610 | 0 | 0 | COPD | No | No | 1 | 1670 | 0 | 0 | 0 | 545 |
| 44 | XLA | 48 | M | 17 | 0 | 720 | 0 | 0 | COPD | No | No | 0 | 3200 | 0 | 0 | 0 | 632 |
| 45 | XLA | 34 | M | 33 | 101 | 830 | 0 | 0 | No | No | No | 0 | 2800 | 0 | 0 | 0 | 399 |
| 46 | XLA | 53 | M | 30 | 30 | 780 | 18 | 24 | COPD | No | No | 4 | 1900 | 0 | 0 | 0 | 453 |
| 47 | XLA | 23 | M | 2 | 0 | 780 | 2 | 3 | No | No | No | 1 | 2050 | 0 | 0 | 0 | 652 |

XLA: X-linked Agammaglobulinemia; CVID: Common Variable Immune Deficiency; M: male; F: Female; CLD: Chronic Lung Disease; LRTI Low Respiratory Tract Infections; CD: Coeliac Disease; ITP: Immune Thrombocytopenia; IBD: Inflammatory Bowel Disease AHE: Hemolytic Anemia; RA: Rheumatoid Arthritis.

**Supplementary Figures**

**Figure S1. Gating strategy to identify RBD+ cells inside total Spike positive (S+ plus S++) MBCs.** Flow cytometry plots in one healthy control and one CVID patient showing the staining pattern of RBD+ MBCs. In the CVID patient, RBD+ MBCs are undetectable.


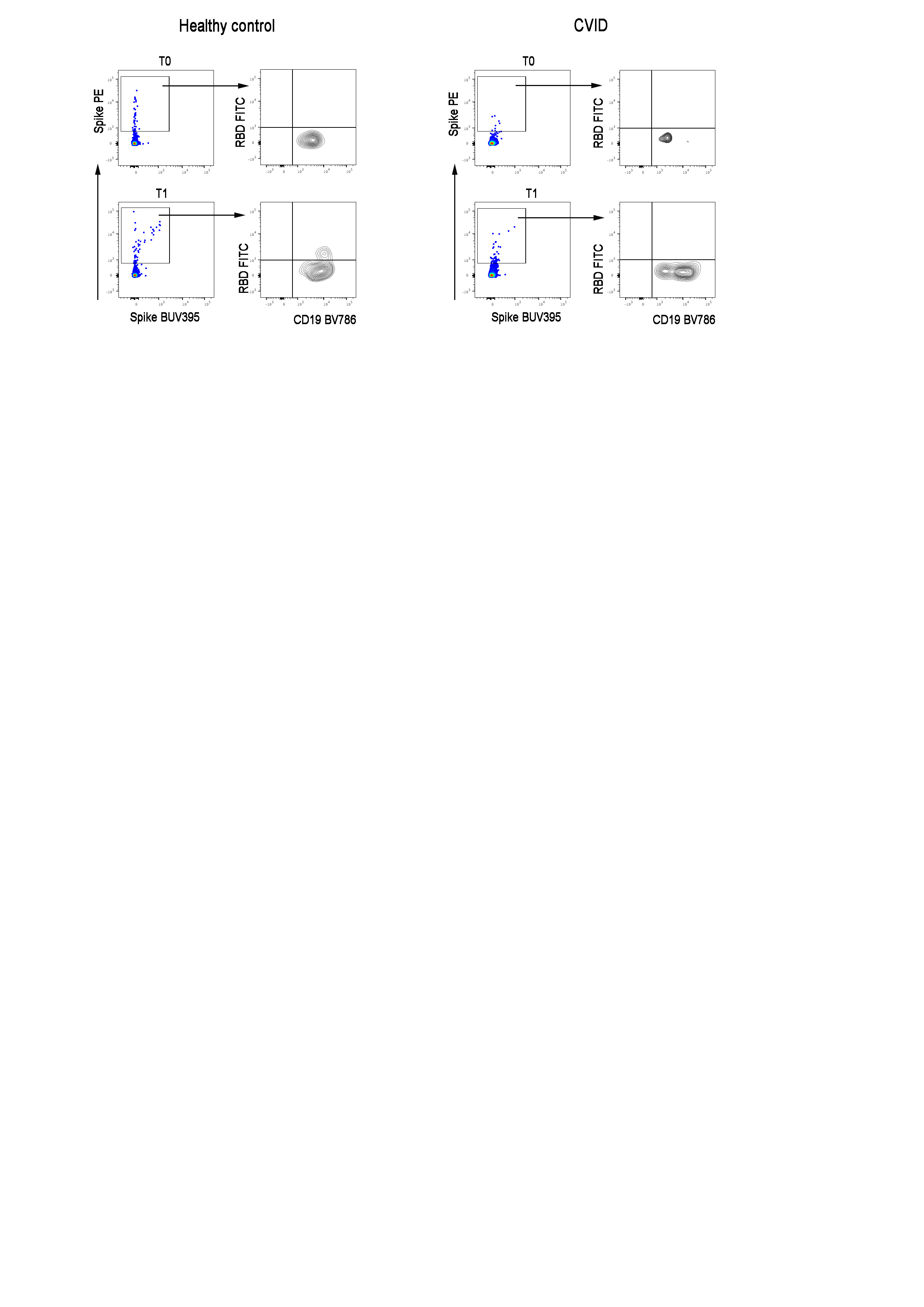


**Figure S2. Identification of IgM+ and IgM- MBCs inside single positive and double positive for recombinant Spike MBCs.** Flow cytometry plots in one healthy control showing the staining pattern. Before immunization S+ are mostly IgM+ and S++ are undetectable. At T1 S+ are still composed by IgM+ MBCs, whereas S++ are IgM-.


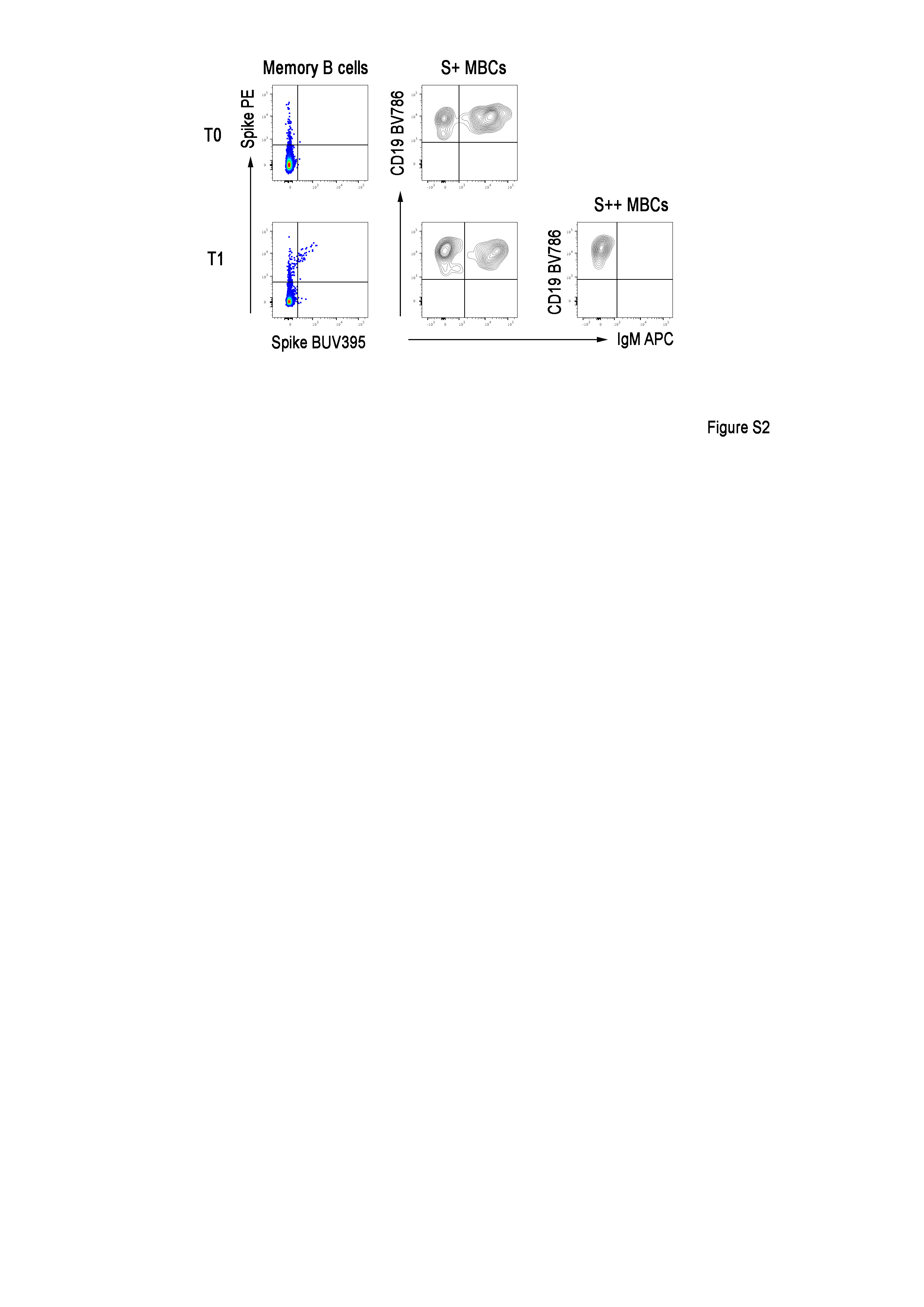

Supplement: Supplementary file 1 — Supplementary file1 (DOCX 853 kb) [file 10875_2021_1133_MOESM1_ESM.docx]
