# Supplementary figures and images for: SARS-CoV-2 Vaccine Induced Atypical Immune Responses in Antibody Defects: Everybody Does their Best
Source: J Clin Immunol. 2021 Oct 20;41(8):1709–22. doi: 10.1007/s10875-021-01133-0 (PMC8527979; doi:10.1007/s10875-021-01133-0)

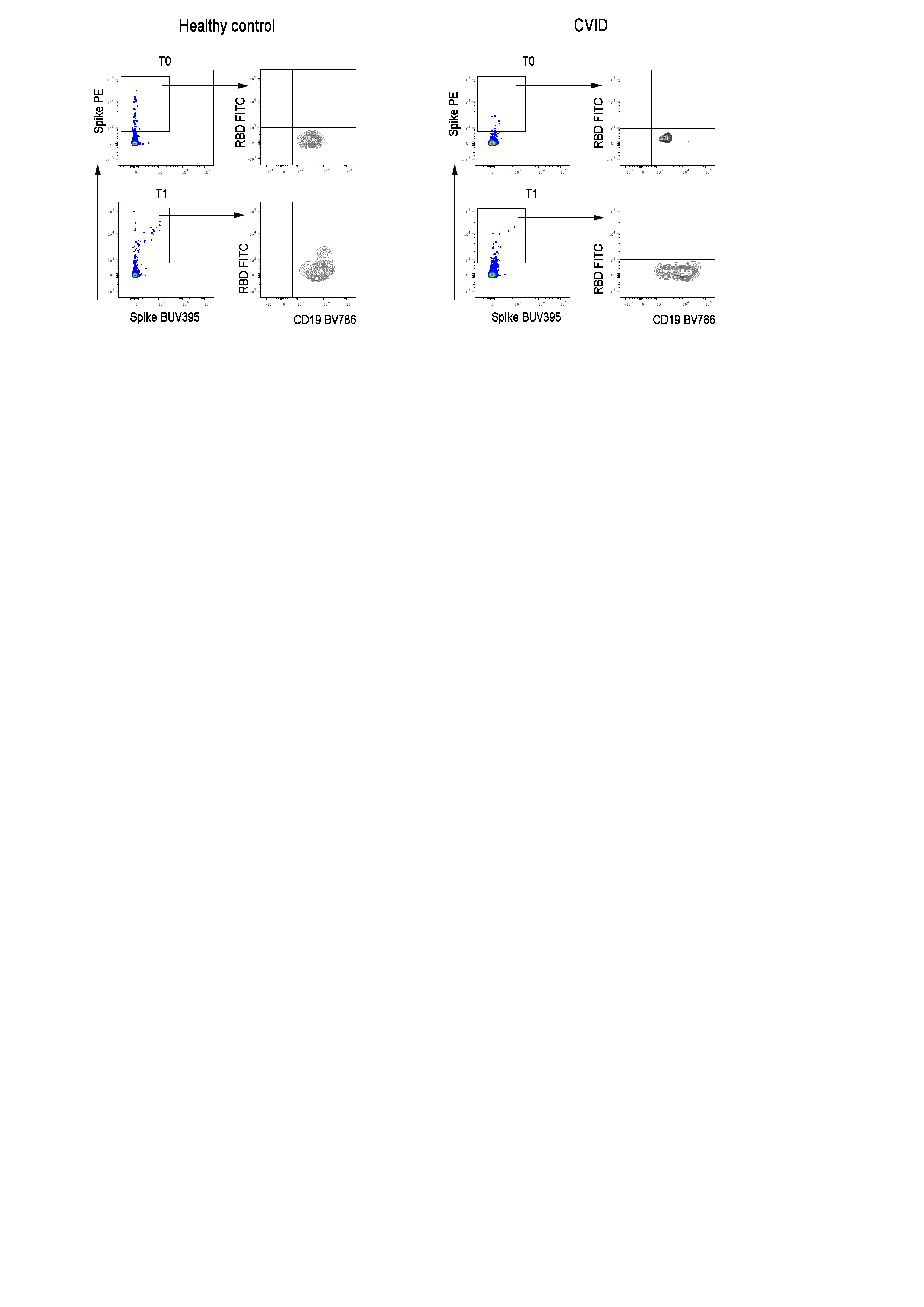

Supplement: Supplementary file 2 — Supplementary file2 (JPG 413 kb) [file 10875_2021_1133_MOESM2_ESM.jpg]

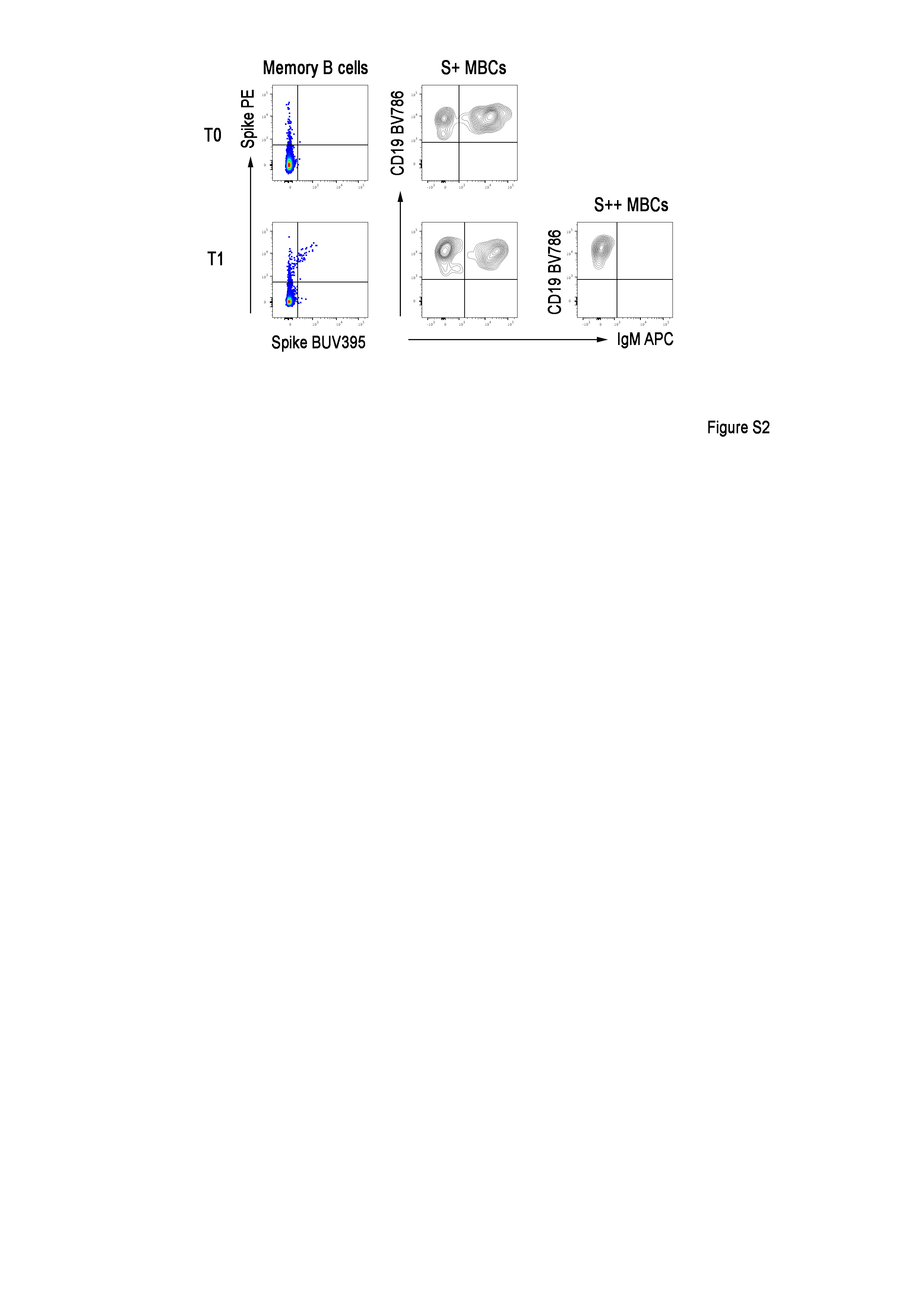

Supplement: Supplementary file 3 — Supplementary file3 (JPG 398 kb) [file 10875_2021_1133_MOESM3_ESM.jpg]
